# Outcome Evaluation in Social Context Measured by Event-Related Potentials Is Partially Dependent on the Partner’s Sex

**DOI:** 10.3389/fpsyg.2018.00853

**Published:** 2018-06-20

**Authors:** Jinping Liu, Shurui Wang, Zhenbiao Jia, Entao Zhang, Mengping Yao

**Affiliations:** ^1^Institute of Psychology and Behavior, Henan University, Kaifeng, China; ^2^Institute of Cognition, Brain and Health, Henan University, Kaifeng, China; ^3^Chongqing Vocational College of Public Transporation, Chongqing, China

**Keywords:** social context, partner sex, outcome evaluation, sex differences, ERP

## Abstract

Outcome evaluation is a cognitive process that people rely on feedback information to evaluate behavior results. It can help people to modify the previous mistakes in order to facilitate the performance of the behavior. In the present study, we examined sex differences in outcome evaluation when men and women performed a “Chuck-A-Luck” dice game with a same-versus opposite-sex partner. We recruited 40 college students (Half of women) to perform the gambling game task, and event-related potentials (ERPs) were recorded for outcome feed back when male or female participants performed the game alone, or with same-versus opposite-sex partners. Two main findings are reported in our study. (1) FRN amplitude of same-sex condition was significantly greater than alone condition for male when the feedback was loss. However, FRN amplitude of opposite-sex condition was significantly greater than alone condition for female when feedback was loss. (2) The loss feedback induced greater P300 than gain only in alone condition. It suggests that sex differences in outcome evaluation is a complex process that is partially influenced by the partner’s sex.

## Introduction

Individuals can quickly and accurately evaluate the valence of feedback information, and modify the previous mistakes in order to facilitate the performance of the behavior, which is called outcome evaluation (Sun and Luo, 2008). It is one of the important functions of the cognitive system. Outcome evaluation can help people monitor the behavior results, detect and correct mistakes in time, adjust the follow-up behaviors and improve the behavior efficiency.

In recent years, the neural mechanism of the outcome evaluation has gradually became a hot topic in the field of cognitive neuroscience (Miltner et al., 1997; Nieuwenhuis et al., 2005). Many studies have traditionally measured the brain activities of outcome evaluation when participants were performing a task alone (Zhou et al., 2010; Luo et al., 2011). However, in daily life, our outcome evaluations are not carried out in isolation, but are modulated by social context, especially by the presence or role of other persons (Leng and Zhou, 2010; Boksem et al., 2011; Yuan et al., 2012; Yilei et al., 2017). For example, Boksem et al. (2011) found that the feedback-related negativity (FRN), reflecting a fast outcome evaluation, was larger when an individual’s own reward was worse than that for others. Unlike the task used by Boksem et al. (2011), using a joint task in which multiple participants performed Chuck-a-Luck dice game together, Li et al. (2010) found that the FRN effect for monetary gains and losses associated with outcomes in joint task became smaller when they played as part of a team compared to when they played alone. These findings have shown that a person’s outcome evaluation is sensitive to the influence of others. More importantly, an outcome is regarded as positive or negative depends on its relevance to the self-interest of the observer (Leng and Zhou, 2010). However, it remains unclear whether there are sex differences in outcome evaluation during two participants performed a task together.

Studies examining sex differences have found important differences between male and female cognition and behavior. Traditionally, women are expected to be more sensitive to social stimuli (Maccoby and Jacklin, 1974). In line with this view, there is some evidence that women tend to pay closer attention to others, including others’ presence, emotions and behaviors, while men tend to focus on their own outcomes over others’ outcomes (Oswald et al., 2004; Mercadillo et al., 2011). However, there is also conflicting evidence for this view (Lithari et al., 2010; Lamm et al., 2011). A recent meta-analytic study indicates that sex differences in social sensitivity depend on contextual factors, for example, increase shared performance is found for male–male groups than female–female groups (Balliet et al., 2011). For mixed-sex condition, other studies have found that individuals perform better while interacting with opposite-sex partners than with same-sex partners (Hirnstein et al., 2014; Cheng et al., 2015). These findings suggest that sex differences in social sensitivity might be modulated by the social context of same- versus opposite-sex partner. Thus, it may be more productive to investigate sex differences in outcome evaluation when men and women perform a task with a same-versus opposite-sex partner.

The present study tested sex differences in outcome evaluation when men and women performed a “Chuck-A-Luck” dice game with a same-versus opposite -sex partner. We used the “Chuck-A-Luck” game in order to create a joint task context in which participants conducted a gambling game alone or co-acting with the same or opposite-sex partners. We adopted electroencephalography (EEG) to access correlated neural activity of outcome evaluation. Two important potentials were analyzed, including the FRN and P300 components. The FRN component is a negative-going deflection that peaks approximately 250–300 ms after the onset of external feedback. It is thought to be a fast and coarse evaluation of external feedback leading to a simple distinction between good and bad outcomes (Hajcak et al., 2005). FRN amplitude has been found to be affected by the extent of self-relevance for the outcome, with larger FRN amplitudes corresponding to higher self-relevance for the outcome (Yeung et al., 2005; Li et al., 2010; Ma et al., 2011). However, the degree to which sex differences affects the FRN remains relatively unexplored. If sex differences in an early outcome evaluation depends on partners’ sex of women and men, we predict a interactive effect between sex and social context in the FRN.

Another ERP component is the P300, which is the most positive peak in the period of 200–600 ms. It has also been found to be related to outcome evaluation or reward processing (Hajcak et al., 2005; Feng et al., 2016). Amplitude of the P300 has also been reported to be sensitive to social contexts, with larger P300 being associated with closer interpersonal distance (Ma et al., 2011). If partner sex could be underlying sex differences in outcome evaluation, we expect to observe sex differences in P300 responses to outcomes, which were modulated by sex of partner.

## Materials and Methods

### Participants

Forty students (mean age 20.5 ± 1.8 years, range 19–24; 20 males) were recruited from the local college. A male participant’s data was deleted, because his EEG is distorted. All participants were right-handed and had normal or corrected-to-normal vision. They were in good health with no previous history of organic brain disorders, and all were experimentally naïve. The experiments were approved by the Henan University Ethics board, and all subjects signed an informed consent form. Subjects were compensated for their participation. All subjects gave written informed consent in accordance with the Declaration of Helsinki.

### Experimental Task Protocol

The joint task consisted of a computerized presentation of a “Chuck-A-Luck” dice game. One 6-sided dice and a blank space representing a second dice are presented to the subject on the computer screen. The subject then attempts to guess whether the result of the second dice being thrown will cause a “win” or a “loss”. If the sum of the two dice on the screen is greater than or equal to 7, the result is a win; less than 7 is a loss. The win/loss result is presented prior to revealing the second dice result. The presentation of stimuli was developed and controlled using E-prime 2.0 professional (Psychology Software Tools, Inc., United States). All images were presented in the white central region of the computer screen on a neutral gray background. Participants were seated in a quiet and electromagnetically shielded room approximately 1 m from a computer screen with the horizontal and vertical visual angles below 5°.

**FIGURE 1:**
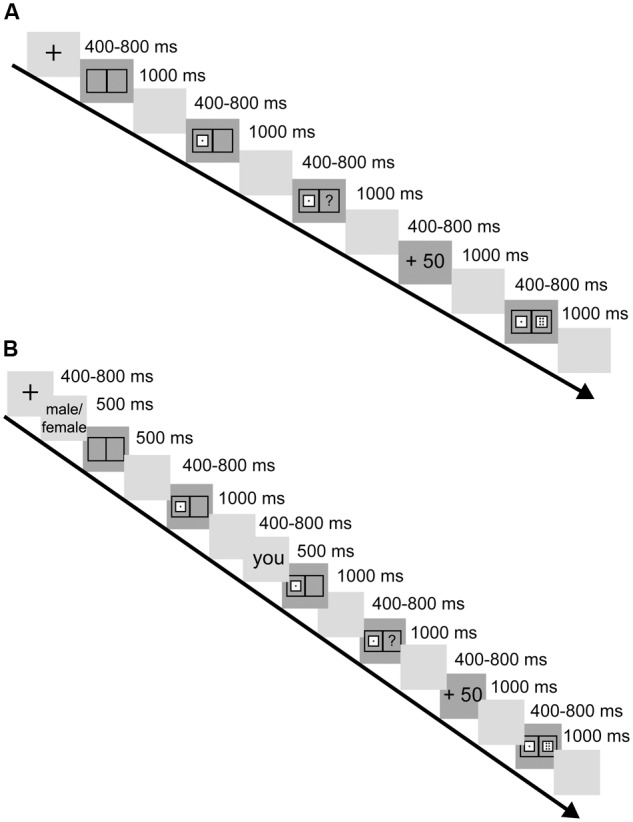
**(A)** Schematic of a trial in the solo block. **(B)** Schematic of a trial within the partner blocks.

The experiment consisted of three blocks, one solo block (see **Figure 1A**) and two partner blocks (see **Figure 1B**). At the beginning of each trial participants were told this task was a simple dice game, and they were to use their index fingers to press the “F” and “J” keys for the left and right frames, respectively. The fixation point was presented in the center of the screen, and participants were told to press F when the frames appeared on the screen. The frames then disappeared, and shortly thereafter a random dice appeared in the left frame and the participants were told to press J. Then the screen blanked again, followed by the appearance of a question mark in the right frame. During this period the participant used the given dice roll to evaluate the probable outcome. After a blank screen, a score was flashed on the screen: “+50” if the result was a win, and “-50” if a loss. Feedback (the score) continued for 800 ms, then after another blank screen, the outcome (the pair of dice) was shown. The screen was blanked and the next trial begun.

For the partner blocks, the participants were seated in the laboratory as instructed by the experimenter. The participants were told prior to beginning the trials that this experiment would be completed together with an unfamiliar partner (the lab assistant). For each trial, the partner would tell the participant that the partner should act first, then press F to begin the dice rolling. When the left frame dice was presented, the participant would press J as described above, and the trial would proceed as described above for the solo block.

Before formal experiment, each participant was given 200 Yuan Token money as the initial funds. According to the results of each trial (lose/win), the number of tokens owned by the subject will be reduced or increased. After the experiment, we exchange subjects’ tokens for cash in appropriate proportions as a reward for participating in the experiment.

### Data Acquisition and Analysis

The EEG was acquired using an elastic cap wired with Ag/AgCl electrodes in the extended 10/20 electrode system, plus two electrodes reserved for electro-oculography (EOG) placed above the left and right eyes. The reference electrodes were behind the right and left mastoid, while the ground electrode was at the midpoint between FP2 and F2. Electrode impedance was kept below 5 KΩ through the experiment. The bandpass was filtered to 0.01–30 Hz, and the sampling rate was 500 Hz. Data were recorded and analyzed via Brain Products ERP (Brain Products GmbH, Germany). Epoch length for offline analysis was 1200 ms including baseline correction; epochs extended 200 ms before stimulus presentation, and continued 1000 ms after presentation. Artifacts from muscle activity with an amplitude ±80 μV or greater were deleted in processing.

### Statistics

Event-related potential values were imported to SPSS 20.0 (IBM, United States). The FRN amplitude can be calculated in two ways, applying grand-averaged waveforms or computing a difference wave (dFRN) between loss and gain trials within 250–350 ms at F3, Fz, F4, FC1, and FC2. And P3 was measured in 350–450 ms at P3, Pz, P4, CP1, and CP2. Two within-subjects factors were used in our studies: joint task type (three levels: alone, same sex, and opposite sex), result (two levels: win and loss).

## Results

### FRN Analysis

**FIGURE 2:**
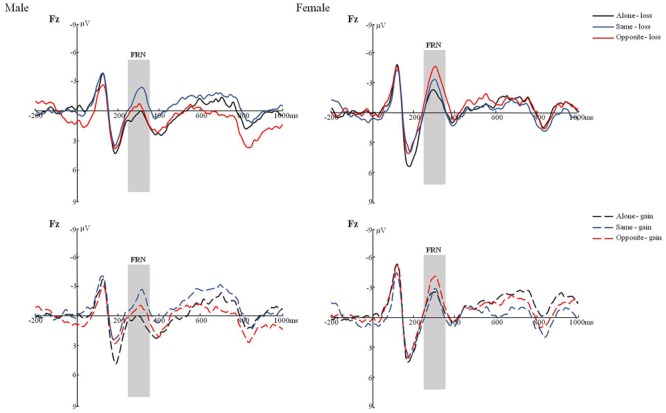
The FRN of male/female under the condition of different joint task in loss/win.

The main effects of Electrode (*F*(4,34) = 18.682, *p* < 0.001, η^2^ = 0.336) was significant. Further comparison found that FRN reached a maximum amplitude appeared at frontal lobe. The interaction effect of Task type, Result, Electrode and gender were significant, *F*(8,30) = 2.710, *p* < 0.05, η^2^ = 0.068. The simple effect analysis revealed that FRN amplitude of same condition was significantly greater than alone condition for male when the result was loss. On electrode F3, *F*(2,17) = 5.61, *p* < 0.01. On electrode F4, *F*(2,17) = 3.23, *p* = 0.051. On electrode Fz, *F*(2,17) = 4.09, *p* < 0.05. On electrode FC1, *F*(2,17) = 4.56, *p* < 0.05, no significant was found on FC2. However, FRN amplitude of opposite condition was significantly greater than alone condition for female when the result was loss. On electrode Fz, *F*(2,18) = 3.82, *p* < 0.05. On electrode FC1, *F*(2,18) = 3.14, *p* = 0.055. No significant was found on F3, F4 and FC2. No gender differences were found, *F* = 0.454, *p* = 0.505 (see **Figure 2**).

**FIGURE 3:**
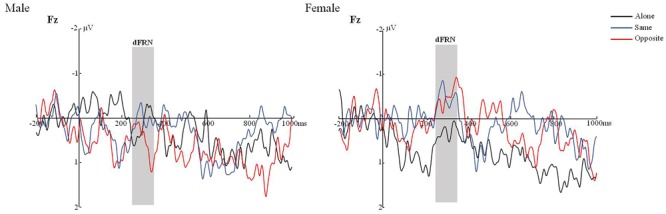
The dFRN of male/female in different joint task conditions.

Previous research suggests additional useful information can be obtained by analyzing the amplitude differentials (Holroyd and Krigolson, 2007; Li et al., 2010), so we analyzed dFRN between the gain and loss conditions via a repeated measures 3 × 5 (Task type × Electrode conditions, see above) ANOVA. The interaction effect of task type, Electrode and gender were significant, *F*(8,30) = 2.710, *p* < 0.05, η^2^ = 0.068. Further analysis found no significant difference. And no gender differences were found, *F* = 0.145, *p* = 0.706. See **Tables 1, 2** and **Figure 3**.

**Table 1:** Results of FRN Analysis.

	*F*	*p*	η^2^
Electrode	18.682	0.000	0.336
Result	0.012	0.913	
Task type	2.857	0.064	
Electrode × Result	0.235	0.918	
Electrode × Task type	0.779	0.622	
Electrode × gender	0.306	0.873	
Result × Task type	1.113	0.334	
Result × gender	0.703	0.407	
Task type × gender	2.429	0.095	
Electrode × Task type × Result	0.720	0.674	
Electrode × Task type × gender	1.396	0.198	
Electrode × Result × gender	0.354	0.841	
Task type × Result × gender	0.087	0.917	
Electrode × Task type × Result × gender	2.710	0.019	0.068

**Table 2:** Results of dFRN Analysis.

	*F*	*P*	η^2^
Electrode	0.235	0.918	
Task type	1.113	0.334	
Electrode × Task type	0.720	0.674	
Electrode × gender	0.354	0.841	
Task type × gender	0.087	0.911	
Electrode × Task type × gender	2.710	0.019	0.068

### P300 Analysis

**FIGURE 4:**
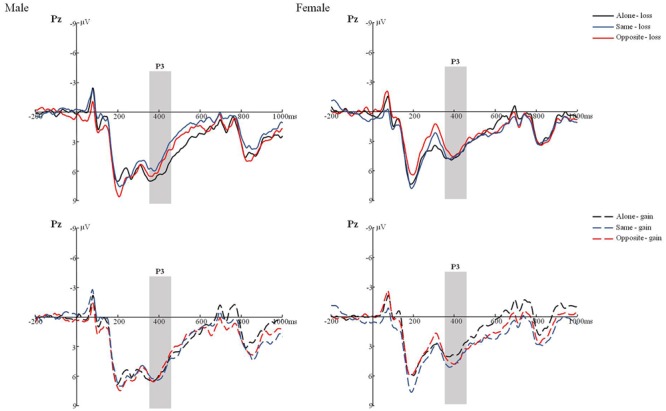
The P3 of male/female under the condition of different joint task in loss/win.

The main effects of Electrode (*F*(4,34) = 15.368, *p* < 0.001, η^2^ = 0.293) was significant. Further comparison found that P3 reached a maximum amplitude appeared at parietal lobe. The Task type × Result interaction was significant, *F*(2,36) = 4.309, *p* < 0.05, η^2^ = 0.104. Further simple effect analysis indicated that result of loss induced greater P300 than gain in alone condition, *F*(1,37) = 3.783, *p* < 0.05, η^2^ = 0.093. This effect was absent in joint conditions (same and opposite). In addition, no other main effects and interactions were significant. No gender differences were found, *F* = 1.088, *p* = 0.304. See **Table 3** and **Figure 4**.

**Table 3:** Results of P3 Analysis.

	*F*	*P*	$\eta_{p}^{2}$
Electrode	15.368	0.001	0.293
Result	0.027	0.869	
Task type	0.074	0.929	
Electrode × Result	1.032	0.393	
Electrode × Task type	0.349	0.946	
Electrode × gender	0.431	0.786	
Result × Task type	4.309	0.017	0.104
Result × gender	7.313	0.541	
Task type × gender	1.084	0.343	
Electrode × Task type × Result	1.767	0.083	
Electrode × Task type × gender	0.562	0.808	
Electrode × Result × gender	0.385	0.819	
Task type × Result × gender	0.802	0.452	
Electrode × Task type × Result × gender	0.469	0.877	

## Discussion

The main result of our experiment is that, for male, the FRN amplitude of same sex condition was significantly greater than alone condition when the result was loss; For female, FRN amplitude of opposite sex condition was significantly greater than alone condition when the result was loss. The data also showed that result of loss induced greater P300 than gain in alone condition. Although the main effects of sex and task context were absent, our results still show that the amplitudes of the FRN and P300 associated with outcome evaluation were partially modulated by partner’s sex and joint task context. Next, we would explain our findings and discuss their implications by comparing these findings with relevant studies.

Our first mainly result was that: For male, the FRN amplitude of same sex condition was significantly greater than alone condition when the result was loss. For female, FRN amplitude of opposite sex condition was significantly greater than alone condition when the result was loss. These findings could be explained in terms of more strong social stress induced by male partner. For a male participant, his same-sex partner was a male, while her opposite-sex was also a male for a female participant. Take together, male partner have shown a stronger social impact both for men and women. There is one possibility that male partner is usually regarded as more powerful than female. However, this explanation should be taken with caution, as there is no direct evidence for this assertion.

Our second finding was that the P300 effect was modulated by task context and the result feedback. The stronger P300 responses following loss feedback compared with win feedback, however, this P300 effect only reach significant in alone condition, and disappeared in joint task conditions (both same and opposite). Given that the P300 is generally thought to be related to processes of attentional allocation or motivational evaluation (Yeung and Sanfey, 2004; Wu and Zhou, 2009; Leng and Zhou, 2010). Our experiments may reflect that difference in motivation between alone condition and joint condition. Responsibility can drive motivation as well. Li et al. (2010) found that a high sense of responsibility was correlated with a larger P300. Nevertheless, Tian et al. (2013) found that electrical components induced by evaluation of results when other people are present could generate larger ERPs than the same evaluation when completing the task alone. Our findings were partially in line with Li et al. (2010), result. This may be the result of motivation being decreased simply by the presence of another.

Besides, we did not find the main effect of task context on the FRN. More specifically, the amplitude of FRN is not affected by the social context in which individuals performed a game with partners. Our result is not consistent with previous studies that smaller FRN effect was found in multiple players joint task (Li et al., 2010; Zhou et al., 2010). The absence of a significant FRN effect in joint task context may reflect the fact that real partners were used in our experiment, whereas just imagined or virtual partners were used in previous studies. The difference of partners could account for the absent FRN effect.

Taken together, during a joint task women and men performed a task with women or men partner to complete a common goal, men partners had more important impacts on their earlier processing of outcome evaluation, which was reflected that FRN effect was larger for men partner condition. However, in a late P300 component, evaluating gambling performance was attenuated by joint task context, and this P300 effect was independent of sex of individual and their partners.

## Conclusion

These findings suggest that an earlier and a later brain responses in outcome evaluation may be modulated by partner’s sex and partner self. Specifically, partner’s sex play a role in the earlier feedback-monitor stage, and partner effect occurs in the late attention-sensitive stage. However, because of joint tasks’ limitation, we can not investigate the real interaction between individual and partners, the most classic ultimatum game may should be taken in future studies. Besides, because of EEG techniques’ limitation, the neural activities were not tracked deeply, FMRI technique would be a good choice in our future studies.

## Author Contributions

EZ and JL designed this study, drafted the manuscript and revised the manuscript. ZJ performed the study and analyzed the data. SW and MY re-conducted the study and analyzed the relevant data.

## Conflict of Interest Statement

The authors declare that the research was conducted in the absence of any commercial or financial relationships that could be construed as a potential conflict of interest.
